# Race and Palliative Care Access in Hospitalized Older Adults with TBI

**DOI:** 10.1093/geroni/igaf122.4245

**Published:** 2025-12-31

**Authors:** Jennifer Albrecht, Chih Chun Tung, Raya Kheirbek

**Affiliations:** University of Maryland, Baltimore, Maryland, United States; University of Maryland, University of Maryland, Maryland, United States; University of Maryland School of Medicine, Baltimore, Maryland, United States

**Keywords:** palliative care, racial disparities, traumatic brain injury

## Abstract

**Background:**

Enhanced understanding of the use of palliative care among older adults with traumatic brain injury (TBI) could help guide development of policy and educational interventions. Our objective was to assess racial and ethnic disparities in the receipt of palliative care among older adults with TBI.

**Methods:**

We conducted a cross-sectional study using data from the Premier Database from May 2022-May 2023. We included adults aged 65 and older with an admission diagnosis of TBI who died during hospitalization. We compared characteristics and palliative care receipt across racial/ethnic groups. Logistic regression models were used to estimate the unadjusted and adjusted odds of receiving palliative care as a function of race/ethnicity. The primary outcome was receipt of a palliative care consultation.

**Results:**

Of 1,119 included patients,76.4% were Non-Hispanic White, 5.1% were Non-Hispanic Black, 5.5% were Hispanic, 4.4% were Asian, and 8.7% were classified as Other/Unknown. The majority (81.7%) received palliative care. In adjusted models, Non-Hispanic Black patients had the lowest odds of receiving a palliative care consultation compared to Non-Hispanic White patients (odds ratio (OR) 0.42; 95% confidence interval, 0.23-0.76).

**Conclusions:**

In a cohort of older adults hospitalized with TBI who died in-hospital, Non-Hispanic Black patients were markedly less likely to receive palliative care compared to their White counterparts. This study underscores the need for future work to identify the extent to which historical mistrust, communication barriers, provider bias, and socioeconomic factors contribute to differences in palliative care access among older TBI patients.

